# Unraveling the Hippocampal Molecular and Cellular Alterations behind Tramadol and Tapentadol Neurobehavioral Toxicity

**DOI:** 10.3390/ph17060796

**Published:** 2024-06-17

**Authors:** Cristiana Soares-Cardoso, Sandra Leal, Susana I. Sá, Rita Dantas-Barros, Ricardo Jorge Dinis-Oliveira, Juliana Faria, Joana Barbosa

**Affiliations:** 1 Associate Laboratory i4HB—Institute for Health and Bioeconomy, University Institute of Health Sciences—CESPU, 4585-116 Gandra, Portugal; cristianacardoso20@outlook.pt (C.S.-C.); sandra.leal@iucs.cespu.pt (S.L.); arbarros2002@gmail.com (R.D.-B.); or ricardo.dinis@iucs.cespu.pt (R.J.D.-O.); 2 UCIBIO—Applied Molecular Biosciences Unit, Translational Toxicology Research Laboratory, University Institute of Health Sciences (1H-TOXRUN, IUCS-CESPU), 4585-116 Gandra, Portugal; 3 UCIBIO—Applied Molecular Biosciences Unit, Toxicologic Pathology Research Laboratory, University Institute of Health Sciences (1H-TOXRUN, IUCS-CESPU), 4585-116 Gandra, Portugal; 4 RISE-HEALTH, Unit of Anatomy, Department of Biomedicine, Faculty of Medicine, University of Porto, Rua Dr. Plácido da Costa, 4200-450 Porto, Portugal; sasusana@med.up.pt; 5 Department of Public Health and Forensic Sciences, and Medical Education, Faculty of Medicine, University of Porto, 4200-319 Porto, Portugal; 6 FOREN-Forensic Science Experts, Av. Dr. Mário Moutinho 33-A, 1400-136 Lisboa, Portugal

**Keywords:** tramadol, tapentadol, prescription opioids, neurobehavioral toxicity, oxidative stress, neuroinflammation, neuromodulation, hippocampal formation, abuse, misuse

## Abstract

Tramadol and tapentadol are chemically related opioids prescribed for the analgesia of moderate to severe pain. Although safer than classical opioids, they are associated with neurotoxicity and behavioral dysfunction, which arise as a concern, considering their central action and growing misuse and abuse. The hippocampal formation is known to participate in memory and learning processes and has been documented to contribute to opioid dependence. Accordingly, the present study assessed molecular and cellular alterations in the hippocampal formation of Wistar rats intraperitoneally administered with 50 mg/kg tramadol or tapentadol for eight alternate days. Alterations were found in serum hydrogen peroxide, cysteine, homocysteine, and dopamine concentrations upon exposure to one or both opioids, as well as in hippocampal 8-hydroxydeoxyguanosine and gene expression levels of a panel of neurotoxicity, neuroinflammation, and neuromodulation biomarkers, assessed through quantitative real-time polymerase chain reaction (qRT-PCR). Immunohistochemical analysis of hippocampal formation sections showed increased glial fibrillary acidic protein (GFAP) and decreased cluster of differentiation 11b (CD11b) protein expression, suggesting opioid-induced astrogliosis and microgliosis. Collectively, the results emphasize the hippocampal neuromodulator effects of tramadol and tapentadol, with potential behavioral implications, underlining the need to prescribe and use both opioids cautiously.

## 1. Introduction

**Figure 1:**
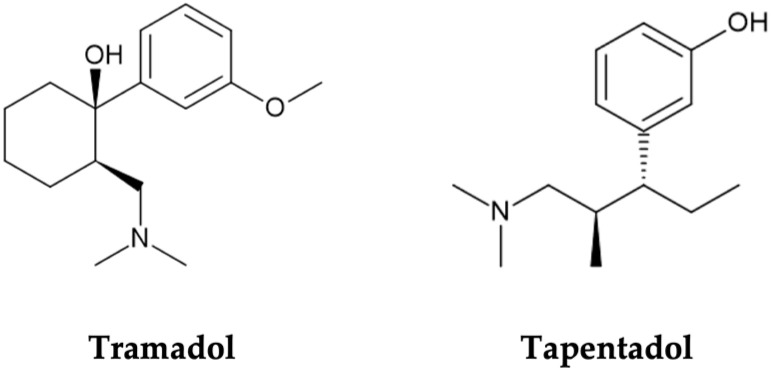
Molecular structures of tramadol and tapentadol, obtained with the ChemSketch software, version 2022.2.2.

Opioids are opium-like drugs commonly used in the treatment of moderate to severe pain, owing to analgesic properties imparted by their activity on µ-, κ-, and δ-opioid receptors, found throughout the central nervous system (CNS) [1,2,3,4]. However, the occurrence of opioid receptors beyond the CNS (e.g., in the gastrointestinal tract) explains some opioid typical adverse events, such as nausea and constipation. To tackle this limitation, new opioid compounds, with additional mechanisms of action, fewer side effects, and a wider therapeutic window, have been designed. Tramadol and tapentadol, two entirely synthetic and atypical opioids, have resulted from this quest for safer opioid options [1,5] (Figure 1).

Similarly to classical opioids (e.g., morphine and codeine), they act as µ-opioid receptor (MOR) agonists; nevertheless, they additionally inhibit monoamine (5-hydroxytryptamine (5-HT) and noradrenaline (NA)) reuptake [1,6,7]. This dual, synergistic mechanism of action is simultaneously responsible for increased analgesia and decreased likelihood of adverse effects and dependence potential [1,5].

Tramadol, (1*RS*, 2*RS*)-2-[(dimethylamino)methyl]-1-(3-methoxyphenyl)-cyclohexanol (Figure 1), is marketed as a racemic mixture [8,9]. Each enantiomer has its own mechanism of action. However, both are required for an effective analgesic effect [1,8,9]. (−)-Tramadol is more effective in NA reuptake inhibition than (+)-tramadol, while the latter is more potent in 5-HT reuptake inhibition [5,7]. (+)-Tramadol also binds MOR more strongly than (−)-tramadol [1,2]. Cytochrome P450 (CYP450) enzymes mainly metabolize the parent drug through *O*- and *N*-demethylation, followed by conjugation reactions [7,10]. Tramadol’s main metabolite, *O*-desmethyltramadol (M1), is the compound with greatest MOR affinity; thus, analgesia is largely dependent on tramadol metabolic bioactivation [5,7]. In turn, tapentadol, 3-[(1*R*,2*R*)-3-(dimethylamino)-1-ethyl-2-methylpropyl]phenol (Figure 1), is a non-racemic compound combining MOR agonism with NA reuptake inhibition while having minimal 5-HT effect, which reduces the risk of developing serotonin syndrome (SS) [1,5]. It is mostly metabolized through phase II reactions, such as glucuronidations and sulphonations [1,5], and has no active metabolites [11,12].

Even though tramadol and tapentadol have succeeded in lowering the occurrence of opioid typical adverse reactions and side effects, dependence, respiratory depression, SS, and fatal intoxications have been reported [1,5]. Due to their considerable CNS activity, neurotoxicity is of particular concern. Numerous in vivo studies have reported altered brain stereological parameters, neuronal degeneration, hypercellularity, apoptotic and irregularly shaped cells, pyknotic cells, red neurons, necroptosis, cytoplasmic and nuclear vacuolization, inflammatory infiltrates, gliosis, microglial and oligodendrocyte proliferation and activation, brain congestion, and edema upon acute to chronic exposure to 25–300 mg/kg tramadol [13,14,15,16,17,18,19,20,21,22,23,24,25,26]. Studies focusing on the analysis of tramadol effects on the hippocampal formation have reported cellular atrophy, shrinkage, necrosis, pyknosis, red neurons, deeply stained and congested vascular channels, as well as pale blue color Nissl granules [27,28].

In fact, behavioral toxicity may be induced by opioids, with tramadol and tapentadol being no exception [29]. Both have led to reports of positive subject-rated effects, such as a sense of happiness, relaxation, decreased anxiety and depression, stimulation, euphoria, emotional liability, as well as other cognitive and mood alterations that might even culminate in hallucinogenic effects at higher doses [5,22,30,31,32,33]. Using a conditioned place preference (CPP) approach, we have shown that tramadol and tapentadol, at their maximum recommended daily dose (50 mg/kg) and for eight alternate days, induce short-term rewarding effects in Wistar rats, with tramadol causing their memory retention [33]. The pre-frontal cortex, amygdala, hippocampal formation, nucleus accumbens, and ventral tegmental area have been implicated in neurotransmission circuits involved in associative learning and memory in dependence, namely in drug reward and reinforcement, cue, and contextual learning [18,34,35,36]. Functional modulation of glutamatergic, GABAergic, and dopaminergic circuits has been related to drug-associated memories, and opioids have been linked to long-term potentiation and depression mechanisms, which are considered the cellular bases of memory formation and learning [18,34,36,37]. Moreover, behavioral disturbances have been linked to alterations in synaptic plasticity induced by neuroinflammation driven by various immune components, such as activated glia, reactive astrocytes, cytokines, chemokines, and reactive oxygen species (ROS), which can impair networks in the mesolimbic system [38,39,40].

Given the profound engagement of the hippocampus in addictive memory and in the retrieval of spatial and contextual associations [34,35,37], we aimed to dissect the molecular and cellular alterations in this brain structure following animal exposure to 50 mg/kg tramadol or tapentadol in the context of CPP assays. The widespread prescription and growing abuse and misuse of both opioids, as well as the sparsity of studies on the neurobehavioral effects of tapentadol, substantiate the pertinence of the present study.

## 2. Results

### 2.1. Repeated Exposure to Tramadol and Tapentadol Causes Oxidative Stress at the Systemic and Hippocampal Levels

Exposure to tramadol and tapentadol has been associated with the induction of oxidative stress, including in the brain cortex [8,13,14]. In fact, acute and chronic exposure to opioids affect redox homeostasis through the generation of reactive oxygen species/reactive nitrogen species (ROS/RNS), which may be directly delivered or produced by activated inflammatory cells [41]. In addition, opioids impair the function of antioxidants (e.g., glutathione (GSH)) and antioxidant enzymes (e.g., catalase, superoxide dismutase, and glutathione peroxidase), as shown in different human and rodent biological matrices, including serum and brain samples [42,43,44]. Morphine, in particular, has been associated with the production of peroxynitrite, formed from superoxide and nitric oxide. Nitric oxide synthase mediates nitric oxide production, whereas nitration and inactivation of spinal manganese superoxide dismutase (MnSOD) leads to peroxynitrite synthesis. NADPH oxidase leads to superoxide generation, with subsequent activation of the phospholipase D pathway and an increase in intracellular Ca^2+^ concentration [43]. Such morphine-induced oxidative stress events have been shown to be mediated by MOR activation [43,45].

**Figure 2:**
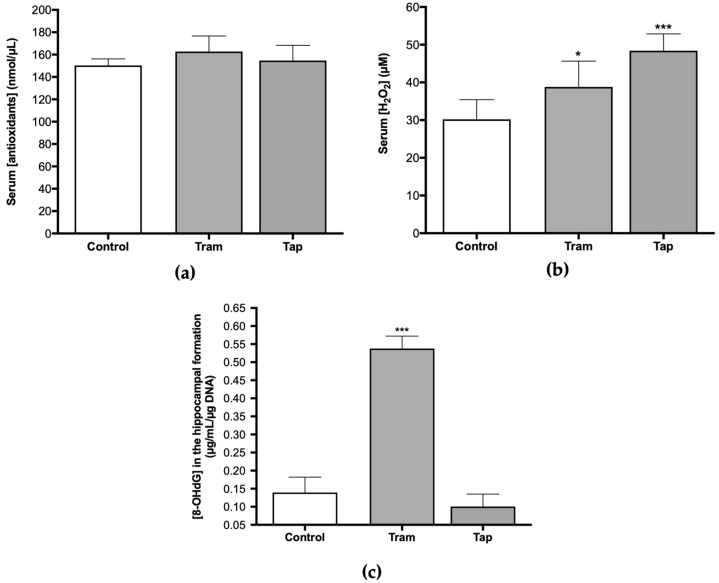
Serum concentration of antioxidants (**a**), H_2_O_2_ (**b**), and hippocampal 8-hydroxydeoxyguanosine (8-OHdG) concentration (**c**) in Wistar rats intraperitoneally administered with 50 mg/kg tramadol (Tram) or tapentadol (Tap), for eight alternate days. 8-OHdG concentration was normalized against DNA content. The results are expressed as means ± SD. * *p* < 0.05; *** *p* < 0.001.

Therefore, analyzing antioxidant defenses and other oxidative stress parameters, such as ROS/RNS, at the systemic and hippocampal levels, after exposure to both opioids, was deemed pertinent. Following exposure to 50 mg/kg tramadol or tapentadol (equivalent to their maximum recommended daily dose) for eight alternate days, no statistically significant changes were observed regarding the serum concentration of antioxidants between the control and the opioid-administered groups (Figure 2a). However, concerning H_2_O_2_ serum concentration, statistically significant differences were observed. There was a 1.28-fold increase in serum H_2_O_2_ concentration in the animals that received tramadol (*p* < 0.05) and a 1.60-fold increase in the group administered with tapentadol (*p* < 0.001), both in comparison with the control group (Figure 2b). Since serum H_2_O_2_ concentration is a surrogate of ROS/RNS levels, it might be deduced that exposure to these opioids, under the conditions of this study, significantly elevates ROS/RNS concentration.

Oral tramadol, administered daily to male albino rats for one month at 25, 50, and 100 mg/kg doses, induces oxidative stress in DNA across various tissues, including the brain, as deduced from the increase in brain 8-hydroxydeoxyguanosine (8-OHdG) levels [17]. In this sense, the concentration of 8-OHdG was assessed in DNA samples isolated from the hippocampal formation of Wistar rats exposed to 50 mg/kg tramadol or tapentadol for eight alternate days. The results indicate statistically significant differences between the tramadol-administered group and the control group (Figure 2c), with a 3.86-fold increase in 8-OHdG concentration in the opioid-administered group in comparison with the controls (*p* < 0.001). No differences were observed when comparing animals exposed to tapentadol with the control group (Figure 2c).

**Figure 3:**
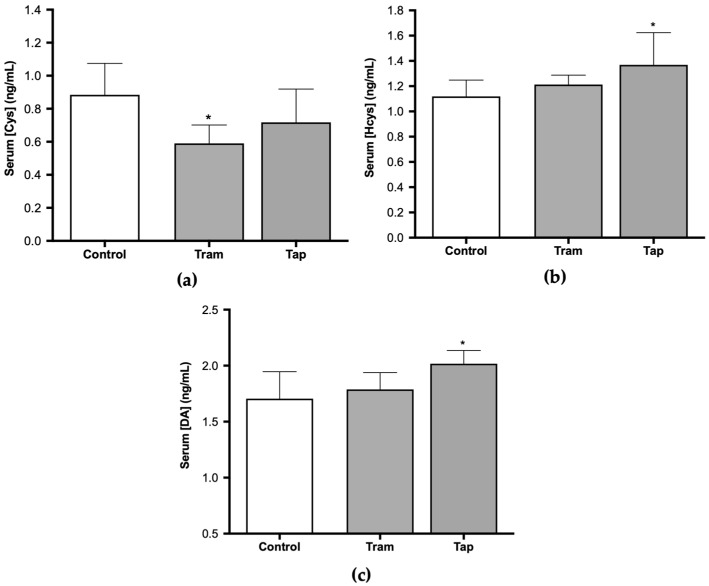
Serum cysteine (Cys) (**a**), homocysteine (Hcys) (**b**), and dopamine (DA) concentrations (**c**) in Wistar rats intraperitoneally administered with 50 mg/kg tramadol (Tram) or tapentadol (Tap) for eight alternate days. The results are expressed as means ± SD. * *p* < 0.05.

Given the antioxidant effect of cysteine (Cys) and the involvement of homocysteine (Hcys) in oxidative stress phenomena [46,47], along with several reports of opioid-induced oxidative stress, the present study also encompassed the quantification of these biomarkers. The analysis of serum Cys and Hcys concentrations in Wistar rats exposed to 50 mg/kg tramadol or tapentadol demonstrated statistically significant differences between the groups. Cys concentration was 67% of the control value in the group administered with tramadol (*p* < 0.05) (Figure 3a). In contrast, a 1.22-fold increase in Hcys concentration was observed in animals administered with tapentadol when compared with the control group (*p* < 0.05) (Figure 3b).

In turn, dopamine (DA) metabolism has been strongly correlated with oxidative stress since its degradation generates ROS, while its oxidation leads to endogenous neurotoxins, and some DA derivatives present antioxidant properties [48]. Moreover, DA release has been associated with dependence on various drugs and substances of abuse. Some authors have presented evidence that tramadol induces an increase in its release [49]. Nevertheless, under the conditions of the present study, DA serum concentration exhibited statistically significant differences for tapentadol-administered animals only, with a 1.18-fold increase in comparison with the control group (*p* < 0.05). No differences were observed between tramadol-treated animals and the control group (Figure 3c).

### 2.2. Repeated Exposure to Tramadol and Tapentadol Affects Neurotoxicity and Neuromodulation Pathways

**Figure 4:**
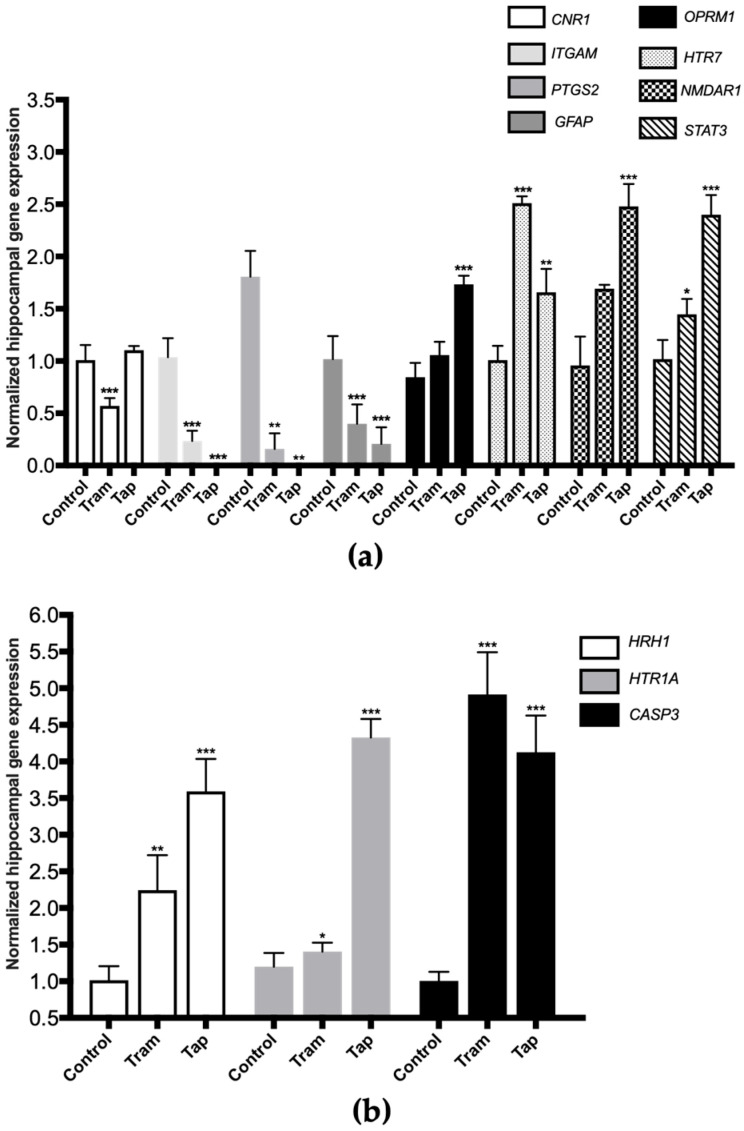
Normalized hippocampal gene expression levels of neurotoxicity and neuromodulation biomarkers upon Wistar rat daily intraperitoneal administration of 50 mg/kg tramadol (Tram) or tapentadol (Tap) for eight alternate days. Gene expression levels were normalized against the respective 18S ribosomal RNA (*18S rRNA*) gene expression and then against the respective controls (administered with normal saline). The results are expressed as means ± SD. * *p* < 0.05; ** *p* < 0.01; *** *p* < 0.001. *CNR1*: cannabinoid receptor 1-encoding gene; *GFAP*: glial fibrillary acidic protein-encoding gene; *HTR7*: 5-hydroxytryptamine receptor 7-encoding gene; *ITGAM*: cluster of differentiation molecule 11b (CD11b)-encoding gene; *NMDAR1*: *N*-methyl-d-aspartate receptor subunit 1-encoding gene; *OPRM1*: µ-opioid receptor 1-encoding gene; *PTGS2*: cyclooxygenase-2 (COX-2)-encoding gene; *STAT3*: signal transducer and transcription activator protein 3-encoding gene (**a**). *CASP3*: caspase-3-encoding gene; *HRH1*: histamine H1 receptor-encoding gene; *HTR1A*: 5-hydroxytryptamine receptor 1A-encoding gene (**b**).

The very same animals analyzed in the present study were previously used in CPP assays, where tramadol and tapentadol have been associated with short-term reinforcing properties; tramadol, in particular, has been shown to cause rewarding memory and incubation of craving [33]. Therefore, the present study also aimed to unravel the molecular alterations underlying such neuromodulation. In this sense, the expression levels of a panel of genes implicated in neurotoxicity and neuromodulation pathways were quantified in hippocampal formation samples from Wistar rats treated with both opioids. To contribute to the characterization of the neuromodulation potential of tramadol and tapentadol, the expression profile of genes involved in neurodegeneration, microglia activation, and neurotransmission was determined in hippocampal formation samples using quantitative real-time polymerase chain reaction (qRT-PCR) (Figure 4). Gene expression levels of opioid-treated animals were compared with those of the control group.

Exposure to 50 mg/kg tramadol for eight alternate days resulted in a statistically significant decrease in the expression of the genes encoding for the following biomarkers: cannabinoid receptor 1 (*CNR1*), cluster of differentiation molecule 11b (CD11b, *ITGAM*), cyclooxygenase-2 (COX-2, *PTGS2*), and glial fibrillary acidic protein (*GFAP*) (decreasing to 57% (*p* < 0.001), 23% (*p* < 0.001), 9% (*p* < 0.001), and 39% (*p* < 0.001) of the control value, respectively) (Figure 4a). In addition, a statistically significant increase was observed in the expression of the genes encoding for 5-hydroxytryptamine receptor 7 (*HTR7*), signal transducer and activator of transcription protein 3 (*STAT3*) (Figure 4a), histamine H1 receptor (*HRH1*), 5-hydroxytryptamine receptor 1A (*HTR1A*) and caspase-3 (*CASP3*) (Figure 4b) (increasing by 2.49- (*p* < 0.001), 1.42- (*p* < 0.05), 2.21- (*p* < 0.01), 1.17- (*p* < 0.05), and 4.89-fold (*p* < 0.001) compared with the control).

Regarding the exposure to 50 mg/kg tapentadol for eight alternate days, a statistically significant decrease was observed in the hippocampal expression of the *ITGAM*, *PTGS2*, and *GFAP* genes (decreasing to 0.8% (*p* < 0.001), 0.03% (*p* < 0.001), and 20% (*p* < 0.001) of the control value, respectively) (Figure 4a). This opioid also induced a statistically significant increase in the expression of the µ-opioid receptor 1 (*OPRM1*), *HTR7*, *N*-methyl-d-aspartate receptor subunit 1 (*NMDAR1*), *STAT3* (Figure 4a), *HRH1*, *HTR1A*, and *CASP3* genes (Figure 4b) (increasing by 2.05- (*p* < 0.001), 1.65- (*p* < 0.01), 2.59- (*p* < 0.001), 2.36- (*p* < 0.001), 3.54- (*p* < 0.001), 4.10- (*p* < 0.001), and 3.61-fold (*p* < 0.001), respectively, compared with the control group).

**Figure 5:**
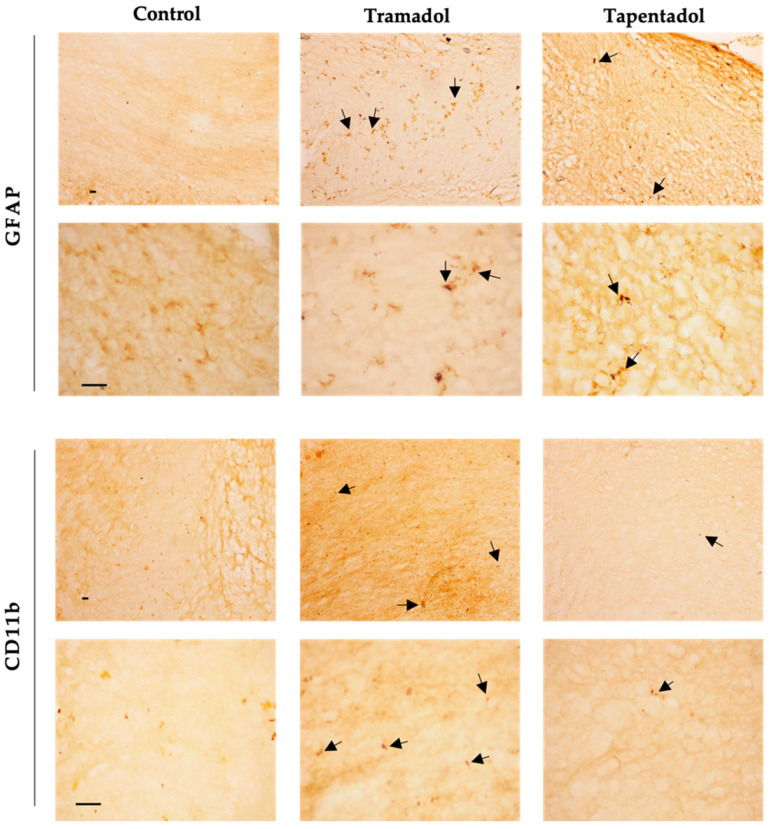
Immunohistochemical staining, with anti-GFAP and anti-CD11b antibodies, of hippocampal formation sections from Wistar rats intraperitoneally administered with 50 mg/kg tramadol or tapentadol or with saline (control group) for eight alternate days. Following opioid exposure, microglial cells presented an ameboid morphology (arrows). Each panel comprises representative photos taken with 100× (**upper figures**) and 400× (**lower figures**) magnifications. Cd11b: cluster of differentiation molecule 11b; GFAP: glial fibrillary acidic protein. Scale bars, 20 µm.

Given the alterations observed in GFAP and CD11b hippocampal gene expression, their protein expression levels were assessed in the same brain structure through immunohistochemistry. Qualitative analysis revealed an increase in the labeling of GFAP, an astrocyte marker, in Wistar rats administered with tramadol and tapentadol compared with the control group. In morphological terms, astrocytes exhibited an ameboid shape, featuring enlarged cell bodies and a notable reduction in their characteristic processes (Figure 5). Regarding CD11b, a microglial biomarker, lower immunoreactivity was observed in the opioid-treated groups compared with the controls. Additionally, microglial cells displayed an ameboid morphology (Figure 5).

## 3. Discussion

Although tramadol and tapentadol, two synthetic analgesic opioids, show a favorable safety profile, they are not devoid of toxicological risks. Numerous adverse reactions, including SS, abuse potential, dependence, and fatal poisonings, have been reported [1,5]. Moreover, their potential for neurobehavioral toxicity phenomena, such as the modulation of reward, stress, memory, learning, and adaptation pathways, must not be disregarded [9,13,33,50]. Previous studies, in in vitro and in vivo contexts, showed neurotoxic damage resulting from single and repeated exposure to therapeutic and supra-therapeutic doses of both opioids [9,13,14,50]. These studies have also reported increased oxidative stress damage in several organs, such as liver and kidney, and identified histopathological changes, including neurodegeneration and microglial activation [9,13,50,51].

In the present study, the increase in ROS/RNS serum levels (Figure 2b) in the animal groups administered with tramadol and tapentadol was consistent with the increase in oxidative stress that is widely reported for different opioids [13,14,50,51,52]. Although a direct comparison is hindered by the use of different study models, exposure periods, routes of administration, opioid doses, and quantification methods, and no statistically different results were detected in SH-SY5Y neuronal cells exposed to 0.25 μM buprenorphine for 48 h, intracellular ROS levels were found to increase by approximately 1.5-fold in cells treated with 10 μM morphine for the same period, as assessed through the 2,7-dichlorofluorescein diacetate (DCFH-DA) assay [45]. An approximate 2.5-fold increase was obtained in DCF fluorescence intensity in the same cell line when exposed to 10 μM morphine for 36 h [53]. Regarding in vivo studies, 4–12 mg/kg morphine, administered for 30 days, led to a 2.5-fold increase in the levels of malondialdehyde (MDA), a lipid peroxidation marker, in rat livers [54]. A 1.7-fold rise in liver MDA was observed upon mice exposure to 20–30 mg/kg morphine for 30 days, while liver protein carbonyl content increased by approximately 1.4-fold, compared with the controls [55]. In rats treated with 5 mg/kg heroin, serum MDA levels rose 1.7-fold above control levels [56]. The magnitude of these alterations is in line with that observed in the present study, for which common oxidative stress mechanisms might be deduced.

Notably, the absence of a concurrent increase in antioxidant serum defenses in the opioid-treated groups suggests a potential for higher oxidative damage, which can directly or indirectly affect the immune function, exacerbate inflammation, and may cause detrimental effects to biomolecules and cell–cell interactions. Astrocytes and microglia react to oxidative stress or pro-inflammatory signals by changing their shape and phenotype from a deactivated state to an activated one [38,57]. Activated microglia perform phagocytic functions, produce ROS, proteolytic enzymes, and an array of pro-inflammatory mediators that alter the activity of non-reactive astrocytes. Intricate interactions between these glial cells and their close association with neurons are necessary to re-establish brain homeostasis and function, which includes repairing the blood–brain barrier, stabilizing extracellular fluid, and reducing the seizure threshold. Conversely, impaired functional capacities of activated astrocytes and microglia can cause disruption of the blood–brain barrier, allowing the infiltration of immune cells, oxidative stress, and inflammation exacerbation. Consistently, an increase in brain cortex thiobarbituric acid-reactive substances (TBARS) and protein carbonyl groups was shown in Wistar rats submitted to daily intraperitoneal (i.p.) doses of 10, 25, and 50 mg/kg tramadol and tapentadol over 14 consecutive days, indicative of oxidative stress damage induced by both drugs [13]. Furthermore, the concentration of 8-OHdG, a biomarker of oxidative DNA damage, exhibited an increase in the hippocampal formation in the tramadol-administered group (Figure 2c). This result suggests the potential genotoxic effects associated with tramadol exposure, which can be explained by an oxidative microenvironment contributed by ameboid-shaped astrocytes and microglia, as observed (Figure 5). Likewise, the decrease in serum Cys concentration observed in animals treated with tramadol (Figure 3a) may be associated with a reduction in antioxidant defenses, particularly GSH, as *N*-acetylcysteine (NAC) is used as a precursor of Cys, a key component in GSH synthesis [46,58]. Although the precise mechanism by which opioids influence Cys concentration is not fully understood, it may be associated with redox-based changes in global DNA methylation and retrotransposon transcription [59]. Additionally, NAC plays a role in the detoxification process of various xenobiotics by reestablishing hepatic GSH levels [58,60]. In this context, previous studies have demonstrated the efficacy of NAC in treating dependence on illicit psychoactive substances [61,62,63]. A study on the effects of melatonin and NAC on oxidative stress, apoptosis, and suppression of blood sexual hormones induced by morphine and methadone in adult male Wistar rats showed that both melatonin and NAC, either alone or in combination, mitigated testicular degeneration, alleviated suppression of sex hormones, and improved testicular function. These beneficial effects were associated with enhanced testicular antioxidant capacity and inhibition of the apoptosis pathway [64]. NAC has also been reported as one of the most effective substances with the ability to counterbalance morphine-induced depletion of GSH, alterations in the activity of antioxidant enzymes, and decreased cell viability [43]. Given the established role of oxidative stress in opioid dependence, tolerance, and addiction, as well as the involvement of redox-based epigenetic changes in drug addiction, treatment with NAC may attenuate behavioral withdrawal symptoms and oxidative stress in opioid dependents [65,66]. Therefore, the decrease in Cys levels observed in the tramadol-treated group could lead to increased oxidative damage and a potential delay in drug detoxification processes, consequently affecting drug effects in the hippocampal formation. These features, combined with dysfunction of astrocytes and microglia in the brain’s reward circuitry, may contribute to memory impairment, as well as to an increased risk of abuse and dependence [39,40,67].

Regarding animals administered with tapentadol, an increase in ROS/RNS serum levels was observed (Figure 2b), which may be related to the induction of oxidative stress mediated by Hcys (Figure 3b) [47,68]. In fact, Hcys has previously been associated with oxidative stress phenomena [47,68], and the present results corroborate this association, showing an increase in serum ROS/RNS levels and glial cells exhibiting an activated morphological phenotype. Moreover, Hcys has been involved in neurodegeneration processes through microglia activation and upregulation of the production of pro-inflammatory cytokines [69,70,71,72]. In this context, elevated Hcys levels result in neuronal damage due to an increase in ROS, a reduction in cytochrome c oxidase activity due to copper chelation, and an increase in pro-apoptotic signals (such as p53, Bax, and caspase-3) [73]. Additionally, elevated Hcys levels continually enhance microglial activation, resulting in a persistent pro-inflammatory condition, ultimately leading to neuronal loss [74]. Furthermore, the amino acid metabolite Hcys activates mTORC1, inhibiting autophagy and leading to the formation of abnormal proteins in human and mice neurons, which is associated with neurodegenerative disorders [75]. Accordingly, studies focusing on Alzheimer’s disease have reported a correlation between high Hcys levels and cognitive decline, associated with microglial activation and increased inflammatory markers within the hippocampal formation [71,76,77]. Moreover, research has demonstrated that Hcys increases glutamatergic activity, leading to excitotoxicity via the activation of NMDA and AMPA receptors [78]. Thus, tapentadol may also be associated with hippocampal vulnerability, induced by disrupted interactions between astrocytes and microglia, contributing to neurotoxic signaling and neuroinflammation. Furthermore, high Hcys levels have been shown to induce neuronal apoptosis and hyperactivate NMDA receptors [79], which is corroborated by the increased expression of the *CASP3* and *NMDAR1* genes in the hippocampal formation of Wistar rats administered with tramadol and tapentadol (Figure 4). Concerning DA, its metabolism has also been associated with oxidative stress. For instance, oxidative deamination by monoamine oxidase (MAO) leads to the generation of hydrogen peroxide, thus contributing to oxidative stress [48]. Consequently, maintaining appropriate levels of DA is crucial to mitigating oxidative stress [48]. In addition, DA is a neurotransmitter involved in several brain circuits and physiological activities, namely movement control, cognition, behavior modulation, and emotion regulation [80,81]. The central neurotransmission of DA is considered a target of addictive substances due to its key role in reward circuits [80,82,83]. In this sense, DA contributes to psychoactive substance dependence [80,82,83]. Evidence from behavioral studies suggests that the release of DA in the nucleus accumbens may be responsible for the rewarding properties of tramadol, highlighting that it might possess a higher abuse potential than previously assumed [49]. In CPP assays conducted by our research team, tapentadol (50 mg/kg i.p.), despite exhibiting significant CNS activity, was shown to have a low propensity to induce memory retention of its rewarding effects when compared with tramadol (50 mg/kg i.p.) [33]. However, tapentadol induced higher preference/reward than tramadol in the short-term [33]. The results of the current study showed an increase in serum DA concentration in animals administered with tapentadol (Figure 3c), suggesting that, at 50 mg/kg, tapentadol may have an increased potential for abuse and dependence. Likewise, increased DA may contribute to the high ROS levels observed in these animals, as previously suggested.

Alterations in the expression levels of a panel of genes implicated in neurotoxicity and neuromodulation pathways were quantified in hippocampal formation samples, aiming to contribute to the elucidation of tramadol and tapentadol effects on such processes (Figure 4). In recent years, there has been a significant focus on understanding the epigenetic signatures associated with opioids. These involve a wide range of chemical modifications in DNA or histone proteins. They dynamically alter the chromatin structure, consequently inducing changes in gene expression in response to opioids [84]. Upon exposure to opioids, alterations in the epigenetic signature occur in both brain and peripheral tissues [84]. Recent findings suggest that opioids stimulate increased levels of permissive histone acetylation and reduced levels of repressive histone methylation. Additionally, opioids induce changes in DNA methylation patterns and the expression of noncoding RNAs across the brain reward circuitry [85]. Notably, methadone, the main maintenance medication for opioid addiction, also induces changes in the DNA methylation status [86]. In fact, multiple in vitro and in vivo studies have demonstrated differential gene expression of inflammation, apoptosis, and autophagy-specific genes in the hippocampal formation and other brain areas following exposure to tramadol [18,22,23]. The hippocampal formation is one of the most important brain areas due to its role in learning, memory, and behavior modulation [67]. Multiple studies have demonstrated negative tramadol effects on learning and memory through MOR activation and induction of neuronal apoptosis [67,87]. Indeed, gene expression analysis in the hippocampal formation, as conducted in the present study, showed a significant upregulation of some genes (Figure 4). The expression of 5-HT1A and 5-HT7 serotonin receptor-encoding genes significantly increased in both groups. In fact, these opioids inhibit serotonin reuptake, thereby prolonging its presence within the synaptic cleft, which may explain the increased expression of serotonin receptors. 5-HT modulates transmission in several physiological and cognitive systems throughout the organism, influencing emotions, memory, sleep, and thermal regulation [88,89]. On the other hand, within the hippocampal formation, these receptors also play a crucial role in learning and memory processes, including reward memory; consequently, their upregulation potentially contributes to the influence of these opioids on the modulation of cognitive processes, as previously suggested by some authors [90]. The expression of the STAT3-encoding gene in the hippocampal formation also increased in both opioid-treated groups, with a more pronounced increase in tapentadol-administered animals. As STAT3 expression is associated with neuroinflammation, neuronal degeneration, and astrogliosis, the observed upregulation suggests that both opioids cause inflammation, with a more pronounced effect for tapentadol [91,92]. In a study conducted with Wistar rats, administered daily through i.p. route with 10, 25, and 50 mg/kg tramadol and tapentadol for 14 consecutive days, both opioids were shown to cause neurodegeneration and led to the accumulation of glial cells in the brain cortex [13]. Furthermore, in a study with a similar experimental design but with acute drug exposure, both prescription opioids were reported to induce neuroinflammation and morphological alterations in neuronal cells, with tapentadol causing greater damage compared with tramadol [14]. The expression of caspase-3, an apoptosis biomarker, showed an increase in both opioid-treated groups, suggesting that tramadol and tapentadol may induce neuronal degeneration through apoptosis as a consequence of neuroinflammation [50,67]. In fact, in a study performed with the human neuronal cell line SH-SY5Y, in which the cells were exposed to 600 µM tramadol and tapentadol, a statistically significant increase in caspase-3 activity was observed in the tapentadol-treated group when compared with the control group [50]. In addition, the upregulation of the histamine H1 receptor-encoding gene was observed in both experimental groups, suggesting the induction of neuroinflammation. This phenomenon could be assigned to the activity of the histamine H1 receptor, promoting the expression of nuclear factor kappa B (NF-κB), a transcription factor that regulates inflammatory processes [93]. Remarkably, the increase was more pronounced in the tapentadol group, suggesting that this opioid has a higher ability to induce neuroinflammation than tramadol. An upregulated expression of the *OPRM1* gene was observed upon exposure to tapentadol, which may be related to its higher potency and higher receptor binding affinity to MOR compared with tramadol [5]. In the literature, changes in the expression of the *OPRM1* gene are reported after exposure to pharmaceuticals and substances of abuse, involving multiple epigenetic mechanisms [94]. The increase observed in the present study is compatible with that reported for chronic fentanyl treatment (in vitro context) and acute oxycodone exposure (in vivo context) [94]. In turn, the NMDA receptor gene was found to be overexpressed in the tapentadol group only, underscoring the influence of this opioid on memory modulation since glutamate, an NMDA receptor agonist, has effects on learning and memory processes, and its release is inhibited by opioids [95]. The NMDA receptor is widely expressed in several brain regions and plays a crucial role in modulating synaptic plasticity and excitatory neurotransmission [96]. Additionally, it is also activated by opioids, as evidenced by the co-localization of MOR and NMDA receptors in some brain regions, which is corroborated by the increased expression of these receptors in the tapentadol-administered group [96].

On the other hand, several genes under analysis showed decreased expression compared with the control group (Figure 4). COX-2, an enzyme associated with prostaglandin synthesis and inflammation, showed decreased expression in both opioid-treated groups [97,98]. COX-2 is constitutively expressed in the brain, particularly in postsynaptic dendrites at the terminals of excitatory neurons [98]. The decrease in COX-2-encoding gene expression could reflect tramadol and tapentadol analgesic efficacy in moderate to severe pain, suggesting reduced inflammation in the hippocampal formation. Additionally, COX-2 interacts with cannabinoid receptors, regulating the formation of ligands that reduce excitatory transmission in the hippocampal formation [98]. The simultaneous decrease in the expression of both COX-2 and cannabinoid receptor 1 suggests that excitatory transmission in the hippocampus is unaffected, consistently with the increased expression of the NMDA receptor-encoding gene. Cannabinoid receptors show high expression in brain regions involved in various cognitive functions, such as memory, learning, regulation of motor activity, emotional behavior, and pain perception [99]. Cannabinoids cause a transient decrease in neurotransmitter release probability and play a significant role in neuromodulation, namely in synaptic plasticity [100,101,102]. The downregulation of the cannabinoid receptor gene expression in the tramadol-treated group suggests that this opioid alters the signaling of the active zone in the presynaptic terminals, consequently affecting synaptic plasticity and reducing neurotransmitter release probability. The expression of the gene encoding for CD11b, a microglia biomarker, is associated with leukocyte adhesion and inflammatory cell migration [103]. As a decrease in the expression of the CD11b-encoding gene was observed in both opioid-treated groups, an immunosuppressive effect might be deduced for tramadol and tapentadol, particularly affecting macrophage-induced phagocytosis. This decrease in gene expression is consistent with the decrease in CD11b protein labeling, as shown by immunohistochemistry assays (Figure 5). Similarly, the expression of the gene encoding for GFAP, an astrocyte biomarker, decreased in both tramadol and tapentadol groups, suggesting a decrease in the hippocampal population of astrocytes, cells that are crucial for the maintenance and regulation of the neuronal environment [16,104]. Owing to their neuronal homeostatic properties, astrocytes have also been related to synaptic plasticity, induction of long-term potentiation, addiction-related memories, and opioid dependence [18,36,37]. Despite the decrease in gene expression, immunohistochemistry assays showed increased GFAP protein levels in the hippocampal formation (Figure 5), suggesting a potential contribution from RNAs from other brain regions to GFAP synthesis. Increased GFAP labeling is consistent with that observed in other studies, in which microgliosis, an increase in the number of glial cells, together with a decrease in the number of neurons, were detected in the striatum and brain cortex of male albino rats following repeated tramadol administration for up to one month [20,22]. Positive GFAP immunostaining was found in association with positive immunoreactivity for p53 (an apoptosis and DNA integrity biomarker) and Iba-1 (a microglia proliferation biomarker) [20,22]. Microglial cells mediate neuroinflammation by promoting the release of pro-inflammatory cytokines, leading to neurodegeneration [20,21]. Overall, our immunohistochemistry analysis revealed increased GFAP and decreased CD11b protein expression, corroborating the evidence of astrogliosis and microgliosis induction by tramadol and tapentadol.

The neuromodulator effects of tramadol and tapentadol, observed upon exposure to a therapeutic dose and for a short period, underline the need for a precautious prescription of both opioids. Accordingly, cautionary recommendations have been made on tramadol and tapentadol use in patients with neurodegenerative diseases, among other special populations [105]. In line with current guidelines, the results from the present study emphasize the importance of sensibly weighing the risks and benefits of opioid prescription; restricting opioid use to situations in which non-pharmacological and nonopioid approaches are ineffective, not tolerated, or contraindicated; beginning treatment with the lowest effective dose, titrating doses gradually, and continuing treatment for the briefest period possible. Periodic reassessment of the risk–benefit ratio on a case-by-case basis is highly advisable, underlining the importance of individualized therapy; if required, changes to therapy may include dose reduction, prolonged dose interval, opioid rotation or discontinuation [105,106,107,108,109].

As a final remark, it should be noted that some aspects could be added to the experimental design of the present work to broaden its scope and depth. The extension of the opioid exposure period beyond that tested in the present study would provide additional data on time-dependent neuromodulator effects exerted by tramadol/tapentadol. Furthermore, by mimicking the chronic use of both opioids, a common situation in clinical practice and in misuse/dependence contexts, such an approach would bring additional realism to the study. The inclusion of additional dosage groups could also offer valuable insights into the dose-effect relationships of tramadol and tapentadol. Since this may provide further mechanistic data concerning the neurobehavioral toxicity and neuromodulation associated with these drugs, it represents a line for future research within the present study’s scope. Lastly, as aforementioned, we have submitted the same Wistar rats for which we document the present molecular and cellular hippocampal alterations to CPP assays [33]. Since these test short-term to long-term and spatial memory, which are known hippocampal functions, the results of the present study have a functional correlation with the neurobehavioral outcomes previously reported by the team. Notwithstanding, given the hippocampal formation involvement in motivational and emotional behavior, memory and learning, behavioral assessments in addition to CPP assays could be carried out in future studies. Examples include operant conditioning, passive avoidance, and T-Y maze tests. These would provide further insights into the link between molecular/cellular alterations and neurobehavioral outcomes, reinforcing the validity of our findings.

## 4. Materials and Methods

### 4.1. Experimental Models and Design

All samples used in the present study were derived from the same animals handled within the scope of CPP assays previously published by the research team [33]. Briefly, twenty-seven male Wistar rats, aged eight weeks, weighing approximately 250 g each, were purchased from the i3S animal facility (Porto, Portugal). Animals were housed, handled, and randomly distributed into three groups (control, tramadol, and tapentadol) of 9 animals each, as reported elsewhere [33]. For 8 days, they received daily i.p. injections of 50 mg/kg tramadol (Sigma-Aldrich, St. Louis, MO, USA) or tapentadol (Deltaclon, Madrid, Spain), alternating with 1 mL saline solution (0.9 g/L (*w*/*v*) NaCl) every other day, following a protocol described by the research team [33]. The dose of tramadol and tapentadol was equivalent to their maximum recommended daily dose for humans and determined using human-into-animal dose conversion, as detailed elsewhere [8,9,13,14,33]. Animals in the control group were administered daily with 1 mL saline solution for 8 consecutive days. Rats were subjected to CPP assays, after which they were sacrificed by i.p. injection of 60 mg/kg sodium thiopental (B. Braun Medical, Queluz de Baixo, Portugal) [33].

As stated in the behavioral study in whose scope rats were handled [33], animal experimentation adhered to the European Council Directive (2010/63/EU) guidelines, incorporated into Portuguese law through Decree-Law no. 113/7 August 2013. Approval for the experiments was granted by the IUCS-CESPU Ethics Committee, Gandra, PRD, Portugal (processes no. PI4AC 2017, PI4AC 2018, PI-3RL 2019 and GI2-CESPU 2022). The National Ethics Council for the Life Sciences (CNECV) guidelines were also observed.

### 4.2. Sample Collection and Processing

Following animal sacrifice, blood samples were drawn through cardiac puncture by using a heparin-containing hypodermic needle. Samples were centrifuged at 3000× *g*, at 4 °C, for 10 min, to obtain serum samples, which were subsequently stored at −20 °C.

Brains were also collected from all animals. Each brain sample was dissected into the right and left hemispheres, which were alternately reserved for biochemical and histological studies, meaning that both studies were carried out with the right and left hemispheres. Regarding the biochemical analysis, the hippocampal formation of each animal was isolated and stored at −80 °C. The other part of the alternate hemispheres was immersed in 4% (*w*/*v*) paraformaldehyde for 24 h and subsequently washed in phosphate buffer saline (PBS: 137 mM NaCl; 2.7 mM KCl; 6.4 mM K_2_HPO_4_; 1 mM NaHPO_4_; pH 7.4) and stored at −80 °C for histological studies.

### 4.3. Quantification of Serum Oxidative Damage Parameters

Total levels of antioxidant agents were quantified in serum samples, diluted 1:30 in ultrapure water, using the Total Antioxidant Capacity Assay Kit (Sigma-Aldrich), according to the manufacturer’s instructions. The protein mask solution was not used, as the aim was to analyze the concentration of small antioxidant molecules and antioxidant proteins. The results were expressed as the concentration of antioxidants (nmol/µL).

Oxidative damage induced by tramadol and tapentadol was quantified in serum samples using the DCF ROS/RNS Assay Kit (Abcam, Cambridge, UK), following the manufacturer’s directions. The assay utilizes an exclusive quenched fluorogenic probe, with fluorescence intensity directly correlating with the overall levels of ROS/RNS present in the sample. Fluorescence results were read in a Synergy 2 plate reader and retrieved using the Gen5, version 1.07.5 software (BioTek, Santa Clara, CA, USA). Serum samples were diluted 1:100 in PBS. Hydrogen peroxide standards were used to plot the calibration curve. The results were expressed as the concentration of H_2_O_2_ (µM).

### 4.4. Quantification of Oxidative Damage Parameters in Hippocampal DNA Samples

DNA samples were extracted from hippocampal formation samples using the NZYol reagent (NZYTech, Lisbon, Portugal), from approximately 10 mg of each sample, following the manufacturer’s directions. Samples were spectrophotometrically analyzed in a NanoDrop™ 2000 (Thermo Scientific, Rockford, IL, USA) to determine DNA concentration and assess protein and organic compound contamination by using optical density (OD) OD_260 nm_/OD_280 nm_ and OD_260 nm_/OD_230 nm_ ratios, respectively. Samples presenting OD ratios ≥ 1.8 were used.

Oxidative DNA damage was evaluated in DNA samples extracted from the hippocampal formation using the OxiSelect™ Oxidative DNA Damage ELISA Kit (8-OHdG Quantitation) (Cell Biolabs, Inc., San Diego, CA, USA), a competitive ELISA assay for the quantitative measurement of 8-OHdG, following the manufacturer’s recommendations. The results were retrieved in a Synergy 2 plate reader, coupled to the Gene5, version 1.07.5 software (BioTek), and expressed as the concentration of 8-OHdG, normalized against DNA content (µg/mL/µg DNA).

### 4.5. Quantification of Serum Biochemical Parameters

Serum Cys, Hcys, and DA concentrations were determined using the Rat Cysteine ELISA Kit, the Rat Homocysteine ELISA Kit, and the Rat Dopamine ELISA Kit, all consisting of competitive ELISA assays, and supplied by AssayGenie (Dublin, Ireland). All determinations were performed with undiluted serum samples and in conformity with all the manufacturer’s specifications. Both samples and standards were analyzed in duplicate. OD values were obtained in a Synergy 2 plate reader coupled to the Gen5, version 1.07.5 software (BioTek). Results were expressed in terms of serum concentrations (ng/mL).

### 4.6. Gene Expression Analysis in the Hippocampal Formation through qRT-PCR

RNA was extracted from hippocampal formation samples using the NZYol reagent (NZYTech), from approximately 10 mg of each sample, and according to the manufacturer’s instructions. The RNA extracted was analyzed in a NanoDrop™ 2000 (Thermo Scientific) to assess its concentration and purity; protein and organic compound contamination were evaluated through OD_260 nm_/OD_280 nm_ and OD_260 nm_/OD_230 nm_ ratios, respectively. Samples with both OD ratios ≥ 1.8 were selected for complementary DNA (cDNA) synthesis. cDNA was synthesized from 400 ng of hippocampal formation RNA using the NZY First-Strand cDNA Synthesis Kit (NZYTech), based on the supplier’s recommendations.

For the qRT-PCR reaction, the NZYSpeedy qPCR Green Master Mix (2×) (NZYTech) was used. Each reaction mixture consisted of 12.5 µL NZYSpeedy qPCR Green Master Mix (2×), forward and reverse primers (STABvida, Caparica, Portugal) to a final concentration of 100 nM each, 10 µL RNase-free water and 2 µL cDNA, diluted 10-fold (equivalent to 4 ng cDNA) in ultrapure water. Each cDNA sample was analyzed in duplicate, totaling 12 replicates for each experimental condition. Primer sequences are detailed in Table 1. Each run incorporated RNA template controls (RTC) and non-template controls (NTC). Plates were analyzed in a C1000 Touch™ thermocycler equipped with the CFX96 Touch Real-Time Detection System (Bio-Rad Laboratories, Hercules, CA, USA), and samples underwent the following reaction program: an initial denaturation for 3 min at 95 °C, followed by 55 cycles of denaturation for 20 s at 94 °C, annealing for 30 s at 55 °C, extension for 30 s at 72 °C, and plate reading. A final melt curve was performed from 65 to 95 °C, with 0.5 °C increments every 5 s, followed by a plate reading step.

Data were retrieved using the CFX Maestro™, version 2.3 software (Bio-Rad Laboratories). The quantification of relative changes in gene expression was performed through the ∆(∆Ct) algorithm, using the *18S rRNA* gene as a housekeeping gene and normalizing results against the control group.

### 4.7. GFAP and CD11b Immunohistochemistry in Hippocampal Formation Sections

Forty µm-thick sections were obtained from brain hemispheres using a vibratome. The sections were serially collected into 24-well plates containing Olmos cryoprotectant solution (0.95% (*w*/*v*) Na_2_HPO_4_·2H_2_O, 0.212% (*w*/*v*) NaH_2_PO_4_·H_2_O, 30% (*w*/*v*) sucrose, 1% (*w*/*v*) polyvinylpyrrolidone (PVP), 30% (*v*/*v*) ethylene glycol), and stored at −20 °C. Samples from 4 animals of each group were analyzed, along with samples from two animals, serving as negative controls, in which all steps were performed except for the incubation with primary antibody. Assays were conducted to analyze labeling with mouse anti-rat GFAP (1:500, BioLegend, San Diego, CA, USA) and mouse anti-rat CD11b (1:500, Millipore, Burlington, MA, USA) antibodies. The Novolink™ Polymer Detection System kit (Leica Biosystems, Deer Park, IL, USA) was used. Slides were observed under a DM500 optical microscope coupled to an ICC50 W camera, and the images were acquired with the LAS V4.13 software, all from Leica Biosystems.

### 4.8. Statistical Analysis

Statistical analysis was performed using an analysis of variance (ANOVA), followed by Dunnett’s multiple comparison test as a post hoc analysis. The results were consistently compared with those of the control group (animals injected daily with saline). The data are presented as means ± standard deviation (SD), and *p*-values < 0.05 were considered statistically significant. Graphic plots and statistical tests were performed using the GraphPad^®^ Prism software, version 9.5.0 (GraphPad Software, LLC, San Diego, CA, USA).

## 5. Conclusions

Taken together, the results from the present study suggest that tramadol and tapentadol cause oxidative stress, inflammation, and neuromodulation in the hippocampal formation, even when administered at therapeutic doses and for short periods. The results concerning tapentadol are particularly relevant, given the scarcity of literature on its adverse events, neuromodulator activity, and underlying molecular and cellular processes.

**Figure 6:**
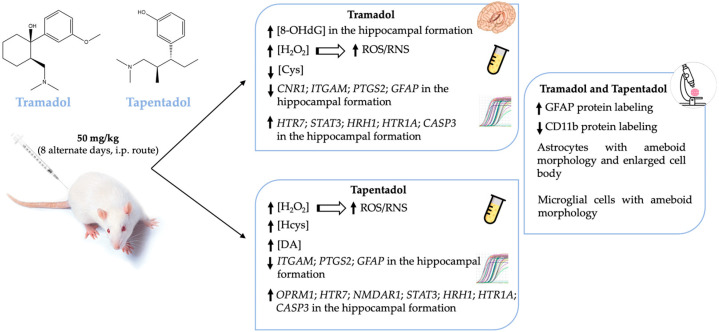
Schematic representation of the main systemic and hippocampal alterations observed in Wistar rats after in vivo exposure to 50 mg/kg tramadol or tapentadol for eight alternate days. *CASP3*: caspase-3-encoding gene; CD11b: cluster of differentiation molecule 11b; *CNR1*: cannabinoid receptor 1-encoding gene; Cys: cysteine; DA: dopamine; GFAP: glial fibrillary acidic protein; *GFAP*: glial fibrillary acidic protein-encoding gene; H_2_O_2_: hydrogen peroxide; Hcys: homocysteine; *HRH1*: histamine H1 receptor-encoding gene; *HTR1A*: 5-hydroxytryptamine receptor 1A-encoding gene; *HTR7*: 5-hydroxytryptamine receptor 7-encoding gene; i.p.: intraperitoneal; *ITGAM*: CD11b-encoding gene; *NMDAR1*: *N*-methyl-d-aspartate receptor subunit 1-encoding gene; 8-OHdG: 8-hydroxydeoxyguanosine; *OPRM1*: µ-opioid receptor 1-encoding gene; *PTGS2*: COX-2-encoding gene; RNS: reactive nitrogen species; ROS: reactive oxygen species; *STAT3*: signal transducer and transcription activator protein 3-encoding gene; ↑: increased levels; ↓: decreased levels.

Treatment with one or both opioids led to alterations in serum H_2_O_2_, Cys, Hcys, and DA concentrations, as well as in hippocampal 8-OHdG and gene expression levels of several neuroinflammation and neuromodulation biomarkers (Figure 6). In turn, increased hippocampal protein expression of GFAP and decreased protein expression of CD11b are consistent with opioid-induced astrogliosis and microgliosis (Figure 6). Such alterations have neurobehavioral toxicity potential and further underline the need to prescribe both opioids conscientiously.

## Figures and Tables

**Table 1:** Genes quantified by qRT-PCR and respective forward (Fw) and reverse (Rev) primer sequences.

Gene	Primer	Nucleotide Sequence (5′ → 3′)	References
*ITGAM* [cluster of differentiation molecule 11b (CD11b)]	Fw	CTGCCTCAGGGATCCGTAAAG	[110]
	Rev	CCTCTGCCTCAGGAATGACATC	
*PTGS2* [cyclooxygenase-2 (COX-2)]	Fw	TGCGATGCTCTTCCGAGCTGTGCT	[111]
	Rev	TCAGGAAGTTCCTTATTTCCTTTC	
*CASP3* (caspase-3)	Fw	GTGGAACTGACGATGATATGGC	[112]
	Rev	CGCAAAGTGACTGGATGAACC	
*GFAP* (glial fibrillary acidic protein)	Fw	CACTCAGTACGAGGCAGTGG-	[113]
	Rev	ACTCAAGGTCGCAGGTCAAG	
*OPRM1* (µ-opioid receptor 1)	Fw	AATCGTCAACGTCTGCAACTGG	[114]
	Rev	GAACGTGAGGGTGCAATCTATGG	
*NMDAR1* (*N*-methyl-d-aspartate receptor subunit 1)	Fw	GCTGTACCTGCTGGACCGCT	[115]
	Rev	GCAGTGTAGGAAGCCACTATGATC	
*HRH1* (histamine H1 receptor)	Fw	CTGGTCACAGTGGGCCTCAA	[116]
	Rev	CTGCCACAGACAGGCTGACAA	
*HTR1A* (5-hydroxytryptamine receptor 1A)	Fw	TCCGACGTGACCTTCAGCTA	[117]
	Rev	GCCAAGGAGCCGATGAGATA	
*HTR7* (5-hydroxytryptamine receptor 7)	Fw	CCGTGAGGCAGAATGGGAAATGTAT	[118]
	Rev	CACTGCGGTGGAGTAGATCGTGTAGC	
*CNR1* (cannabinoid receptor 1)	Fw	AGGAGCAAGGACCTGAGACA	[119]
	Rev	TAACGGTGCTCTTGATGCAG	
*STAT3* (signal transducer and activator of transcription protein 3)	Fw	CAAAGAAAACATGGCCGGCA	[120]
	Rev	GGGGGCTTTGTGCTTAGGAT	
*18S rRNA* (18S subunit of ribosomal RNA)	Fw	GCAATTATTCCCCATGAACG	[121,122]
	Rev	GGCCTCACTAAACCATCCAA	

## Data Availability

Data are contained within the article.
